# Pyruvate production using engineered *Escherichia coli*

**DOI:** 10.1186/s13568-016-0259-z

**Published:** 2016-10-08

**Authors:** Hironaga Akita, Nobutaka Nakashima, Tamotsu Hoshino

**Affiliations:** 1 Research Institute for Sustainable Chemistry, National Institute of Advanced Industrial Science and Technology (AIST), 3-11-32 Kagamiyama, Higashi-Hiroshima, Hiroshima 739-0046 Japan; 2 Bioproduction Research Institute, National Institute of Advanced Industrial Science and Technology (AIST), 2-17-2-1 Tsukisamu-Higashi, Toyohira-ku, Sapporo, 062-8517 Japan; 3 Department of Biological Information, Graduate School of Bioscience and Biotechnology, Tokyo Institute of Technology, 2-12-1-M6-5 Ookayama, Meguro-ku, Tokyo, 152-8550 Japan

**Keywords:** Pyruvate, *Escherichia coli*, Promoter regulation, Tetracycline, Doxycycline, Fermenter

## Abstract

Pyruvate plays an essential role in the central carbon metabolism of multiple organisms and is used as a raw material in the chemical, biochemical and pharmaceutical industries. To meet demand, large amounts of pyruvate are produced through fermentation processes. Here we describe a simple and efficient method for producing pyruvate in *Escherichia coli*. To stop carbon flux from pyruvate to fatty acids, the *accBC* genes, which encode the enzyme that catalyzes the first step of fatty acid biosynthesis and is essential for vegetative growth, were manipulated within the genome; its native promoter was replaced with the tetracycline (or doxycycline)-regulated promoter and the corresponding transcriptional regulator genes. The resulting strain grew normally in the presence of doxycycline, but showed poor growth upon withdrawal of doxycycline. Using this strain, we developed a high pyruvate producing strain (strain LAFCPCPt-accBC-aceE), in which the tetracycline-regulated promoter was also introduced upstream of *aceE*, and the *ackA*-*pta*, *adhE*, *cra*, *ldhA*, *pflB* and *poxB* genes were deleted. After determining the optimal culture conditions for this strain, the final pyruvate concentration reached 26.1 g L^−1^ after 72 h with a theoretical yield of 55.6 %. These levels are high enough to indicate that the developed strain has the potential for application to industrial production of pyruvate.

## Introduction

Pyruvate is a key metabolite in such catabolic and anabolic pathways as glycolysis, gluconeogenesis, and amino and fatty acid metabolism. In addition, commercial demand for pyruvate has been expanding every year. In biochemical industries, for example, pyruvate is used as a building block for the synthesis of antioxidants (Wang et al. 2007), food additives (EFSA 2009), and dietary and weight control supplements (Kalman et al. 1999; Saper et al. 2004). Pyruvate is also used as a raw material in the pharmaceutical industry. For example, various pharmaceutical precursors, including *N*-acetyl-d-neuraminic acid (Zhang et al. 2010), l-3,4-dihydroxyphenylalanine (Park et al. 1998) and *R*-phenylacetylcarbiol (Rosche et al. 2001) are produced from pyruvate.

**Fig. 1:**
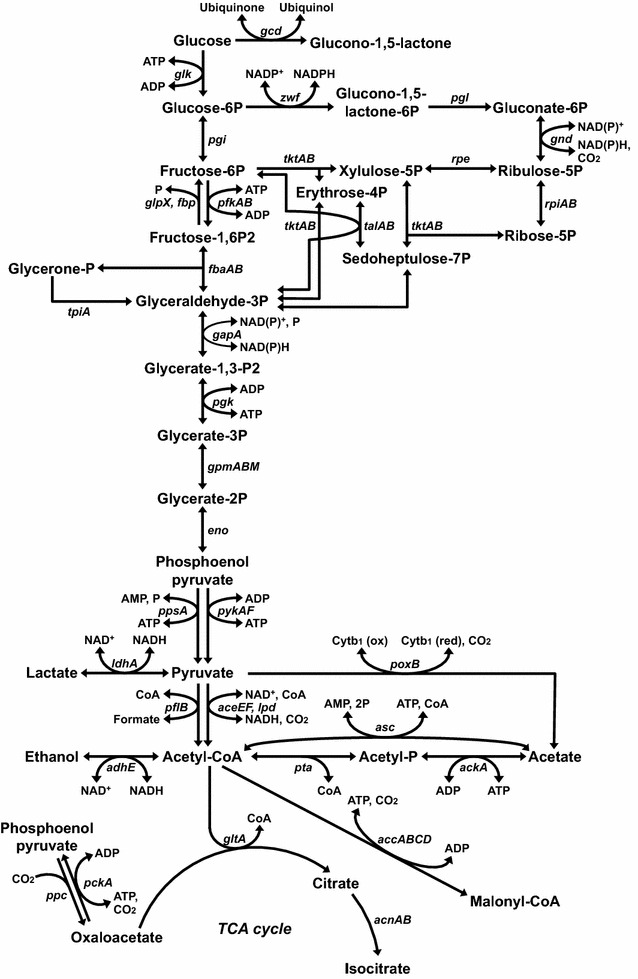
Glucose metabolism in *Escherichia coli*

Commercial methods of pyruvate production can be roughly classified as chemical, enzymatic or fermentation processes (Li et al. 2001a). In the chemical process, pyruvate is synthesized mainly through dehydration and decarboxylation of tartrate. But while this method is easy to implement, it is not cost effective (Li et al. 2001a). Compared with the chemical process, the enzymatic process effectively reduces the production cost and improves yield (Li et al. 2001a). The fermentation process is the most widely used in industry, because it is sustainable, cost-effective and achieves high yields and productivity (Li et al. 2001a; Xu et al. 2008). For the fermentation process, a multi-vitamin auxotroph strain of *Torulopsis glabrata* is primarily used (Li et al. 2001a). With *T. glabrata* strains, a pyruvate concentration of more than 40 g L^−1^ was reached (Li et al. 2001a), though maintenance of the concentration balance among biotin, nicotinic acid, pyridoxine and thiamine was required (Li et al. 2001b). Consequently, the availability of this method is limited by the necessity for special expertise and expensive equipment. On the other hand, a lipoic acid auxotroph strain of *Escherichia coli* has also developed as a producer (Table 1). For example, *E. coli* CGSG 7916 showed high pyruvate accumulation in controlled fermentations (Tomar et al. 2003). This strain was constructed through *aceF* mutation. The *aceEF* and *lpd* genes express the pyruvate dehydrogenase complex, which catalyzes conversion of pyruvate into acetyl-CoA (Fig. 1; CaJacob et al. 1985). Thus, pyruvate accumulation was achieved by decreasing the activity of the pyruvate dehydrogenase complex. In addition, *E. coli* YYC202, an *aceEF* deletion strain, showed excellent pyruvate productivity (Zelić et al. 2003). However, this strain required acetate for growth due to its weak capacity for acetyl-CoA production. *E. coli* TBLA-1 was constructed by transduction of a F1-ATPase-defective gene into *E. coli* W1485*lip2*, which is a derivative strain from *E. coli* K-12 (Yokota et al. 1994). This mutation enhanced both glucose consumption and pyruvate production. *E. coli* TC44 was constructed through decreasing the ATP yield, cell growth and CO_2_ production as well as deletion of the acetate, ethanol and lactate production pathways (Causey et al. 2004). During *E. coli* TC44 fermentations, changing the oxygen saturation from 5 to 50 % enhanced the pyruvate productivity. In *E. coli* TC44, mutation of the *poxB* gene, which encodes pyruvate oxidase, was most beneficial for growth and pyruvate production, as the mutation enhanced the NAD^+^ concentration in the cells and activated several enzymes involved in the glycolysis pathway. Thus construction of *E. coli* strains for pyruvate production involves introducing mutations that reduce utilization of pyruvate for cell growth and deletion of nonessential pathways through pyruvate metabolism.

**Table 1:** Comparison of the pyruvate productivities of *E. coli* strains

Strain	Genotype	Carbon source	Concentrations (g L^−1^)	Productivity (g L^−1^ h^−1^)	Yield^a^ (%)	References
*E. coli* LAFCPCPt-accBC-aceE	MG(1655) Δ*ldhA* Δ*adhE* Δ*pflB* Δ*pta-ackA* Δ*poxB* Δ*cra* P*tet*-*accBC* P*tet*-*aceE*	Glucose	4.2^b^	0.0584	10.6	This study
*E. coli* LAFCPCPt-accBC-aceE	MG(1655) Δ*ldhA* Δ*adhE* Δ*pflB* Δ*pta-ackA* Δ*poxB* Δ*cra* P*tet*-*accBC* P*tet*-*aceE*	Glucose	26.1^c^	0.363	55.6	This study
*E. coli* CGSG (6162)	F^+^ *aceF*10 *fadR*200 *tyrT*58(AS) *adhE*80 *mel*-1	Glucose, acetate	37.0	1.03	ND	Tomar et al. (2003)
*E. coli* CGSG (7916)	CGSC(6162) *ppc*::Kan	Glucose, acetate	35.0	0.972	ND	Tomar et al. (2003)
*E. coli* TC44	W(3110) (Succ^+^), Δ*focA-pflB*::*FRT* Δ*frdBC* Δ*ldhA* Δ*atp(FH)*::*FRT* Δ*adhE*::*FRT* Δ*sucA*::*FRT* Δ*poxB*::*FRT* Δ*ackA*::*FRT*	Glucose	52.0	1.21	77.9	Causey et al. (2004)
*E. coli* W(148*5*)*lip2*	W(1485) F^+^ λ^−^ *lipA2*	Glucose	25.5	0.797	52.2	Yokota et al. (1994)
*E. coli* TBLA-1	W(148*5*)*lip2 bgl* ^+^ *atpA401*	Glucose	31.5	0.984	64.4	Yokota et al. (1994)
*E. coli* YYC202	Hfr *zbi*::Tn*10 poxB1* Δ(*aceEF*) *rpsL pps-4 pfl-1*	Glucose, acetate	62.0	1.75	ND	Zelić et al. (2003)
*E. coli* ALS929	YYC202 *ldhA*::Kan	Glucose, acetate	70.0	2.06	ND	Zhu et al. (2008)
*E. coli* ALS(1059)	YYC202 *ldhA*::Kan *arcA726*::FRT *atpFH*::Cam	Glucose, acetate	90	2.05	ND	Zhu et al. (2008)

*ND* not described

^a^Yield was the theoretical maximum value

^b^Pyruvate was produced from N5G medium under standard culture conditions for 72 h

^c^Pyruvate was produced from N5G medium under optimized culture conditions for 72 h

Here we report an effective method for using promoter regulation for pyruvate production. To control essential gene expressions, tetracycline-regulated promoter (P*tet*) was inserted upstream of the *accBC* genes, and gene expressions were regulated by the absence of doxycycline. After the pyruvate producing strain was constructed, optimal culture conditions for pyruvate production were determined.

## Materials and methods

### General genetic techniques

*E. coli* strain MG1655 (wild-type, The Coli Genetic Stock Center; CGSG6300) and its derivative strain LAF was described earlier (Nakashima and Tamura 2012). Gene deletion and knock-in were carried out as described earlier (Emmerson et al. 2006; Nakashima et al. 2014a, b; Nakashima and Miyazaki 2014). The sequences of all primers are shown in Table 2.

**Table 2:** PCR primers for construction of plasmids

Primers	Sequences
sSN1185	5′-ATCCATGGTCGTCTCACTCGCGAACTGC-3′
sSN1186	5′-GTGGATCCCCAGATGCGTTTCACCCCTGC-3′
sSN1187	5′-AATCTAGAACAGGCCAAAGGTTTCAGCCTG-3′
sSN1188	5′-GATATGCATCGCCACTTTATCCAGCGGTAG-3′
sSN1892-accN	5′-AAACTGCAGGGGTGAATAATCTGATTTTGTTTGACTAC-3′
sSN1893-accN	5′-AAATCTAGACAGGCGTTTCACCGCCGTCTGTAAAGC-3′
sSN1894-tetR	5′-TTACTAGTTTAAGACCCACTTTCACATTTAAGTTGTTTTTC-3′
sSN1895-tetR	5′-AAAGGATCCTTTCTCCTCTTTAATGAATTCGGTCAGTG-3′
sSN1896-accORF	5′-AAAGGATCCATGGATATTCGTAAGATTAAAAAACTG-3′
sSN1897-accORF	5′-CCGACATGTTCAGATAACTTTTTACTGACGGAGC-3′
sSN1997aceEDox1	5′-TCAACATGTCTAACTACTTCAATACACTGAATCTG-3′
sSN1998aceEDox2	5′-AGACTAGTTTAACCCCCCAGTTTCGATTTATCGCG-3′
sSN1998aceEDox3	5′-AAAATGCATAGGCCTTCTCGGGCATAAGTCGGACACCATGG-3′
sSN1999aceEDox4	5′-ATTGCGAGGCTTTGTGCTTCTCTGG-3′
sSN2000aceEDox5	5′-CCCATGGGGATCCAGGCCTTCTAGAACT-3′
sSN2001aceEDox6	5′-GGGGTACCTGAGCAAACTGGCCTCA-3′

The restriction enzyme site is underlined

Unless stated otherwise, *E. coli* cells were cultured in Luria–Bertani medium at 37 °C. To evaluate cell growth, culture pre-grown overnight was diluted 1:500, and doxycycline was added if necessary. Cell density was measured at 600 nm in 200 µL aliquots of culture in a 96-well plate (Nalge Nunc International, Rochester, NY, USA; product no. 269,620) using a Safire microplate reader (Tecan, Männedorf, Switzerland) and a LS-PLATEmanager 2004 data analysis program (Wako, Osaka, Japan).

### Construction of a plasmid for deletion of *poxB*

The 5′- and 3′-flanking regions of *poxB* were PCR-amplified from genomic DNA from strain MG1655 using specific primer sets: sSN1185/sSN1186 and sSN1187/sSN1188, respectively. The 5′ end of sSN1185 was phosphorylated using T4 DNA kinase before use. The termini of the amplified fragments were treated with *Nco*I and *Nsi*I, respectively, and the two fragments were cloned into the *Pst*I–*Nco*I site of pHN1234, yielding pHN1243. Plasmid pHN1243 was used for disruption of *poxB*.

### Construction of plasmids for knock-in of tetracycline promoters

A DNA fragment containing the 5′-flanking region of *accBC* was PCR-amplified using primers sSN1892-accN and sSN1893-accN. The amplified fragment was then cut with *Pst*I and *Xba*I and cloned into the *Pst*I–*Xba*I site of pHN1234 (Nakashima and Tamura 2012), yielding pHN2125. A DNA fragment containing P*tet* and transcriptional regulator gene (*tetR*) was PCR-amplified using primers sSN1894-tetR and sSN1895-tetR from pHN1271 (Nakashima and Tamura 2013). That amplified fragment was cut with *Spe*I and *Bam*HI and cloned into the *Spe*I–*Bam*HI site of pHN2125, yielding pHN2127. A DNA fragment containing the 3′-flanking region of *accBC* was PCR-amplified using primers sSN1896-accORF and sSN1897-accORF. The amplified fragment was then cut with *Bam*HI and *Nco*I and cloned into the *Bam*HI–*Nco*I site of pHN2127, yielding pHN2128. Plasmid pHN2128 was used to knock in P*tet* and *tetR* into the upstream of *accBC*.

To introduce P*tet* upstream of *aceE* within the genome, pHN2198 was constructed. A DNA fragment containing the 5′-flanking region of *aceE* was PCR-amplified using primers sSN1997aceEDox1 and sSN1998aceEDox2. The amplified fragment was then cut with *Pst*I and *Xba*I and cloned into the *Pst*I–*Xba*I site of pHN1234 (Nakashima and Tamura 2012), yielding pHN2187. A DNA fragment containing P*tet* was PCR-amplified using primers sSN1998aceEDox3 and sSN1999aceEDox4, with pHN1271 serving as a template. The amplified fragment was then cut with *Spe*I and *Pst*I and ligated into the *Spe*I–*Bam*HI site of pBluScriptII KS(+) using T4 DNA ligase, yielding pHN2189. A DNA fragment containing the 3′-flanking region of *aceE* was PCR-amplified using primers sSN2000aceEDox5 and sSN2001aceEDox6, after which the amplified fragment was cut with *Pst*I and *Eco*RV and cloned into the *Pst*I–*Eco*RV site of pHN2189, yielding pHN2191. A DNA fragment cut from pHN2191 using *Spe*I and *Nco*I was cloned into the *Xba*I–*Nco*I site of pHN2187, yielding pHN2198.

### Standard culture conditions for pyruvate production

Strain LAFCPCPt-accBC-aceE was pre-grown overnight in Luria–Bertani medium and then diluted 1:100 with fresh N5G medium (pH 7.2) containing 60 g L^−1^ glucose, 10 g L^−1^ (NH_4_)_2_SO_4_, 2 g L^−1^ NaCl, 1 g L^−1^ KH_2_PO_4_, 0.24 g L^−1^ MgSO_4_·7H_2_O and 0.011 g L^−1^ CaCl_2_·2H_2_O. Pyruvate production was performed in a small-scale multistage fermentor Bio Jr. 8 (Able &amp; Biott, Tokyo, Japan) with a working volume of 80 mL. The standard culture conditions were as follows: culture temperature, 37 °C; culture pH, 7.2; stirrer speed, 1200 rpm; airflow rate, 50 mL min^−1^.

### Optimization of the culture conditions

The effect of culture temperature was examined at 29–37 °C. The culture pH was tested at pH 5.4–7.2 through automatic addition of 4 M NaOH solution. The stirrer speed and airflow rate were examined at 1200–2000 rpm and 50–150 mL min^−1^, respectively. The OD_600_ was measured by monitoring the difference between the cell and cell-free turbidity values using an Eppendorf BioSpectrometer (Eppendorf, Hamburg, Germany).

### Quantification of pyruvate and glucose

After clarifying the culture by centrifugation and filtration, pyruvate and glucose were quantified using a high performance liquid chromatograph equipped with a Jasco UV-2070 Plus Intelligent UV/VIS Detector at 210 nm (Jasco, Tokyo, Japan), a Jasco RI-2031 Plus Intelligent Refractive Index Detector (Jasco) and an Aminex HPX-87H cationic exchange column connected to an Aminex 85H Micro-Guard Column (Bio-Rad Labs, Hercules, CA, USA). The chromatographic conditions were as follows: mobile phase, 4 mM H_2_SO_4_; flow rate, 0.5 mL min^−1^; column oven temperature, 65 °C.

## Results

### Control of *accBC* expressions by the tetracycline-regulated promoter

In *E. coli* cells, the *accABCD* genes are annotated as essential genes for growth. The *accABCD* gene products comprise the acetyl-CoA carboxylase complex, which catalyzes the biotin-dependent carboxylation of acetyl-CoA to produce malonyl-CoA via two half-reactions (Broussard et al. 2013). In the first half-reaction, biotin carboxylase, which is encoded by *accC*, catalyzes the ATP-dependent carboxylation of biotin. Immediately after the carboxylation, the carboxylated biotin is attached to the biotin carboxyl carrier protein, which is encoded by *accB*, resulting in the synthesis of carboxy-biotin. In the second half-reaction, carboxyltransferase, which is encoded by *accAD*, transfers the carboxyl group from carboxy-biotin to acetyl-CoA, yielding malonyl-CoA. In an earlier study, we observed that silencing *aceE* expression increased the accumulation of pyruvate (Nakashima et al. 2014b). In addition, we also reported that silencing both *accA* and *aceE* led to even greater pyruvate accumulation (Nakashima et al. 2014b). Those results indicate that pyruvate accumulation is achieved by significantly decreasing the activities of both the pyruvate dehydrogenase complex and the acetyl-CoA carboxylase complex. In the present study, therefore, we used P*tet*, which is controlled by the presence or absence of doxycycline, to regulate the expressions of *accBC* and *aceE*.

**Fig. 2:**
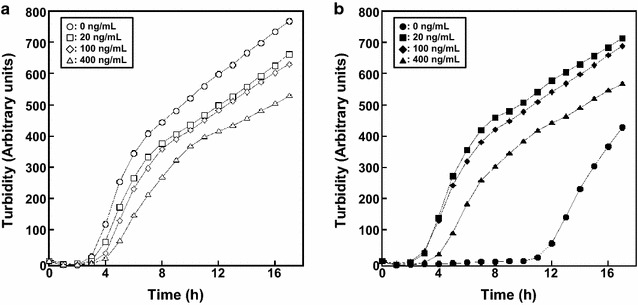
Monitoring growth of wild type (**a**) and *E. coli* strain Pt-accBC (**b**). *E. coli* strains were incubated at 37 °C in Luria–Bertani medium containing doxycycline at concentrations of 0, 20, 100 and 400 ng mL^−1^, as indicated

To confirm the effect of P*tet* on gene expression, we assessed our ability to control *accBC* expressions. When doxycycline was present in the culture, the growth curves obtained with strain Pt-accBC were similar to those obtained with the wild-type strain (Fig. 2). On the other hand, growth of strain Pt-accBC was retarded in the absence of doxycycline (Fig. 2b). These results demonstrate that P*tet* is useful for controlling essential gene expression.

### Development of a pyruvate producing strain

In *E. coli* cells, acetate is produced via two primary routes (Fig. 1): (1) conversion of acetyl-CoA to acetate by phosphotransacetylase (*pta*) and acetate kinase (*ackA*) (Kakuda et al. 1994) and (2) oxidation of pyruvate to acetate by pyruvate oxidase (*poxB*) (Abdel-Hamid et al. 2001). To block acetate production from pyruvate in *E. coli* cells, the *ackA*–*pta* and *poxB* genes were deleted.

The Cra protein, global transcriptional regulator, controls transcriptional expression of genes involved in sugar catabolism (Saier and Ramseier 1996). Moreover, we previously reported that disruption of *cra* was also beneficial for accumulating pyruvate in *E. coli* cells, since this disruption led to a more rapid rate of pyruvate production (Nakashima et al. 2014b). Therefore, *cra* was also deleted. The resultant strain, LAFCPCPt-accBC-aceE, was then used for experimentation.

### Pyruvate production using standard and optimized conditions

**Fig. 3:**
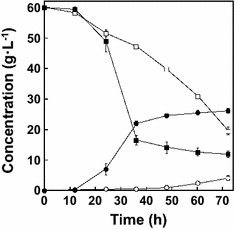
Pyruvate production under standard and optimized culture conditions. The strains were cultured in N5G medium. Shown are the time-dependent changes in pyruvate (*circles*) and glucose (*squares*) concentrations under standard (*open symbols*) and optimized (*filled symbols*) culture conditions. *Error bars* indicate SE (*n* = 3)

When 60 g L^−1^ glucose was provided as the carbon source under the standard culture conditions (see “Materials and methods” section), strain LAFCPCPt-accBC-aceE pyruvate levels reached 4.2 g L^−1^ in 72 h with consumption of 40.5 g L^−1^ glucose (Fig. 3).

**Fig. 4:**
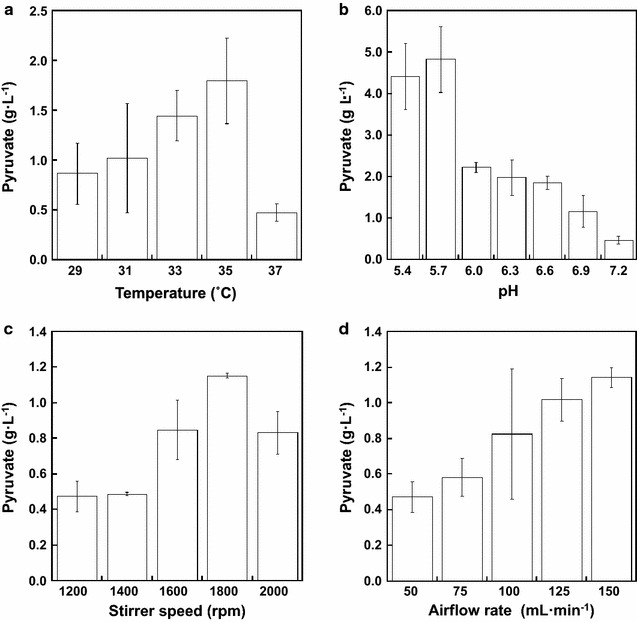
Effects of culture conditions on pyruvate production. *E. coli* strain LAFCPCPt-accBC-aceE was cultivated for 24 h in N5G medium. Shown are the effects of temperature (**a**), culture pH (**b**), stirrer speed (**c**), and airflow rate (**d**) on pyruvate production. *Error bars* indicate SE (*n* = 3)

**Fig. 5:**
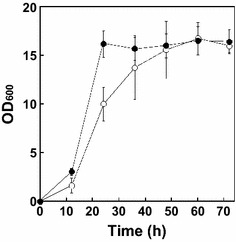
Growth of *E. coli* strain LAFCPCPt-accBC-aceE under standard and optimized culture conditions. The strains were cultured in N5G medium under standard (*open symbols*) and optimized (*filled symbols*) culture conditions. *Error bars* indicate SE (*n* = 3)

To optimize the culture conditions for pyruvate production by strain LAFCPCPt-accBC-aceE, the effects of temperature, initial culture pH, stirrer speed and airflow rate were evaluated. The maximum pyruvate concentration was obtained at 35 °C (Fig. 4a). When the effect of initial culture pH was assessed, the highest product concentration was obtained at pH 5.7 (Fig. 4b). When the stirrer speed was investigated, the highest final product concentration was obtained at 1800 rpm (Fig. 4c). Finally, examination of the airflow rate conditions showed that 150 mL min^−1^ produced the highest product concentration (Fig. 4d). In sum, the optimal culture conditions for pyruvate production were as follows; culture temperature, 35 °C; culture pH, 5.7; stirrer speed, 1800 rpm; airflow rate, 150 mL min^−1^. Under these optimized conditions, the pyruvate concentration reached 26.1 g L^−1^ after 72 h with consumption of 48.0 g L^−1^ glucose (Fig. 3). Moreover, the productivity was 6.2-fold higher than was achieved under the standard conditions, and the theoretical yield was 5.2-fold higher. Also, strain LAFCPCPt-accBC-aceE showed improved growth rate under optimized culture conditions (Fig. 5).

## Discussion

In this study, we demonstrated that P*tet* was useful for controlling essential gene expressions. Subsequently, we have constructed a high pyruvate producing strain LAFCPCPt-accBC-aceE. After optimal culture conditions for pyruvate production were determined, the final pyruvate concentration reached 26.1 g L^−1^ after 72 h with a theoretical yield of 55.6 %. In our knowledge, this yield was similar compared with the yields of another engineered *E. coli* strains (Table 1). However, our pyruvate producing strain was not required supplemental carbon additives and special expertise for pyruvate production, which was easy to perform. These results indicate that strain LAFCPCPt-accBC-aceE also has an industrial potential for pyruvate production.

With pyruvate production using strain LAFCPCPt-accBC-aceE, we think the culture pH is the most important factor. pH is known to have a significant effect on gene expression in *E. coli* cells, and expression of *aceE* is promoted at acidic pH (Maurer et al. 2005). On the other hand, after deletion of the *ackA*–*pta* pathway, cell growth is accelerated at acidic pH and the activity of the *poxB* gene product is enhanced (Dittrich et al. 2005). Similarly, pyruvate productivity in a *poxB*-deleted strain is higher at acidic pH than neutral pH (Dittrich et al. 2005). Taken together, these observations suggest that both the production and degradation of pyruvate is stimulated at acidic pH, and thus pH 5.7 was optimal in our study. Note that pyruvate degradation is limited in our study due to gene manipulation.

We also observed that both stirrer speed and airflow rate influenced pyruvate production. Transcription of several genes involved in the gluconeogenesis and anaplerosis pathways (*pckA*, *ppsA*, *ppc* and *sfcA*), the TCA cycle (*gltA*), the glyoxylate cycle (*aceA*), and acetate metabolic pathways (*acs*, *ackA*, *pta* and *poxB*) are affected by the dissolved oxygen concentration (Phue and Shiloach 2005). In our study, more aerobic conditions showed improved growth rate (Fig. 5), presumably to support facilitate overall carbon flux (Matsuoka and Shimizu 2013).

We found that the optimal culture temperature was 35 °C (Fig. 3a), though the reason is unclear. This temperature has no effect on expression of the genes involved in glycolysis or the pentose phosphate pathway (Gadgil et al. 2005). Perhaps this temperature contributes to enhancing the growth rate. For example, when *E. coli* strain ML30G was cultured in glucose minimal medium at various temperatures, the maximum growth rate was observed at 35 °C (Shehata and Marr 1975).

## Abbreviations

P*tet*: tetracycline-regulated promoter
*tetR*: transcriptional regulator gene
